# Complete Mitochondrial Genome Sequence of *Acrida cinerea* (Acrididae: Orthoptera) and Comparative Analysis of Mitochondrial Genomes in Orthoptera

**DOI:** 10.1155/2010/319486

**Published:** 2010-12-06

**Authors:** Nian Liu, Yuan Huang

**Affiliations:** College of Life Sciences, Shaanxi Normal University, 199 South Chang'an Road, Xi'an, Shaanxi 710062, China

## Abstract

The complete 15,599-bp mitogenome of *Acrida cinerea* was determined and compared with that of the other 20 orthopterans. It displays characteristic gene content, genome organization, nucleotide composition, and codon usage found in other Caelifera mitogenomes. Comparison of 21 orthopteran sequences revealed that the tRNAs encoded by the H-strand appear more conserved than those by the L-stand. All tRNAs form the typical clover-leaf structure except *trnS* (*agn*), and most of the size variation among tRNAs stemmed from the length variation in the arm and loop of TΨC and the loop of DHU. The derived secondary structure models of the *rrnS* and *rrnL* from 21 orthoptera species closely resemble those from other insects on CRW except a considerably enlarged loop of helix 1399 of *rrnS* in Caelifera, which is a potentially autapomorphy of Caelifera. In the A+T-rich region, tandem repeats are not only conserved in the closely related mitogenome but also share some conserved motifs in the same subfamily. A stem-loop structure, 16 bp or longer, is likely to be involved in replication initiation in Caelifera and Grylloidea. A long T-stretch (>17 bp) with conserved stem-loop structure next to *rrnS* on the H-strand, bounded by a purine at either end, exists in the three species from Tettigoniidae.

## 1. Introduction

Mitochondrial genomes exhibit several unique features, including strict orthology, maternal inheritance, lack of recombination, and rapid evolutionary rate. Due to key technological advances in sequencing and the accumulation of universal primers, mitochondria genes have been routinely used in phylogenetic studies as molecular markers [1]. In insect, the mitogenome is a double-stranded circular DNA molecule, usually composed of 13 protein coding genes (*cox1-3*, *cob*, *nad1-6*, *nad4L*, *atp6,* and *atp8*), 22 transfer RNA genes (*trnX*, where *X* refers to the corresponding amino acid), and 2 ribosomal RNA genes (*rrnS* and *rrnL*, respectively). In addition, an embedded large A+T-rich noncoding region may contain signals for control of replication and transcription. In certain metazoans mtDNA, all genes are transcribed from one strand, whereas in others both strands are used. Except for tRNA encoding genes, the gene order of entire mitochondrial genomes appears to be highly conserved in insects [2, 3].

For phylogenetic reconstruction, the entire mitogenome sequences contain more information than simply the collection of individual gene sequences. Examination of the mitogenomes may reveal important genome-level characteristics, such as length variation, base compositional bias, codon usage, gene rearrangement, RNA secondary structures, and modes of control of replication and transcription [4]. Gene rearrangements have become a very powerful means for inferring ancient evolutionary relationships, since rearrangements appear to be unique, generally rare events that are unlikely to arise independently in separate evolutionary lineages. Rearrangements have been found in over a third of the insect orders and in those orders where multiple representatives have been examined the phylogenetic signal in rearrangements is often very strong. Nevertheless, Mitogenome rearrangements have not lived up to early promise as useful phylogenetic markers for the resolution of interordinal relationship. The majority of insects have the same plesiomorphic gene arrangement that is shared by the Pancrustacea [2, 5, 6]. 

As the secondary structure of RNA (rRNA) molecules is considerably conserved across distantly related taxa, the structural information helps to refine the alignment of rRNA sequences more accurately in phylogenetic analyses [1, 7–11]. Although the secondary structure models have proliferated over the past decades in conjunction with the increasing number of molecular phylogenetic studies based on rRNA sequences, details of mitochondrial rRNA structure are still usefully investigated because they may differ even among closely related taxon in peripheral regions [8]. Likewise, advances in RNA substitution models have underlined the need for reliable secondary structure models for individual taxonomic groups [12]. 

The control region is called the A+T-rich region in insect, which is the major noncoding region in the mitogenome of insect [1]. It is heavily biased to A+T nucleotides and seems to evolve under a strong directional mutation pressure. Among insects, this region is variable in both size and nucleotide sequence and may contain tandem repetition which is often associated with heteroplasmy. In contrast, the nucleotide substitution rate in this region is likely to be much reduced due to high A+T content and directional mutation pressure [13]. Some structural elements, which have been proposed to be involved in the control of replication and transcription, have been observed to be highly conserved between phylogenetically very distant insect taxa. These observations have implications for the use of this region as a genetic marker in evolutionary studies [13–15]. Therefore, comparison of mitogenomes at various taxonomic levels may result in significant insights into the evolution of both organisms and genomes.

Orthoptera is a group of large and easily recognizable insects which includes grasshoppers, locusts, ground hoppers, crickets, bush-crickets, and mole-crickets as well as some lesser known groups. It is divided into two suborders: Caelifera and Ensifera, with ~20,000 known species distributed around the world. Most grasshoppers are herbivorous, often regarded as agricultural pests. *Acrida cinerea*, commonly known as the Chinese grasshopper, belongs to the subfamily Acridinae in Acrididae. The genus *Acrida* comprises approximately 40 species, occurred in Africa, Europe, Asia, and Australia. In China, 8 *Acrida* species are found and *A. cinerea* is the most widely distributed [16]. The grasshoppers of the genus *Acrida* are omnivorous insects, which are well known to damage sorghum, wheat, rice, cotton, weed, sweet potato, sugar cane, Chinese cabbage, or other crops. 

51 sequence entries from this subfamily have been listed in the GenBank and most of them are partial mtDNA sequences of *Acrida*. Fenn et al. [17] presented the complete mitogenomes of *Acrida willemsei* and other four orthopteran species. The paper reconstructed a preliminary phylogeny of Orthoptera as a vehicle to examine the phylogenetic utility of mitogenome data in resolving deep relationships within the order. They also explored various methods of analyzing mitogenome data in a phylogenetic framework, by testing the effects of different optimality criteria, data partitioning strategies, and data transformation. 

Here, the complete mitogenome of *A. cinerea* (Acrididae: Orthoptera) was reported with emphasized common structure elements and variations of RNA molecules and A+T-rich region based on the comparative sequence analyses with other 20 orthopterans. Hopefully these efforts would be helpful to understand the evolution characterization of mitogenome structure of orthopteran and provide basic structural information for RNA sequence alignment for evolution and phylogenetic studies in future.

## 2. Materials and Methods

### 2.1. Sampling

*A. cinerea *specimens were collected from Taibai Mountain at Xi'an, Shaanxi, China. All specimens were preserved in 100% ethanol and stored at −4°C.

### 2.2. DNA Extraction, PCR, and Sequencing

Total genomic DNA was isolated from a female adult* A. cinerea* by phenol/chloroform method and was diluted to 50 ng/*μ*l in double-distilled water and used as template for long and accurate polymerase chain reaction (LA-PCR). 

**Figure 1:**
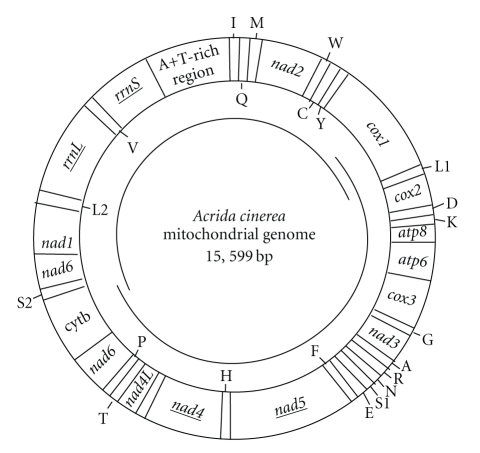
Gene map of the *A*.* cinerea* mitogenome. Protein coding genes are transcribed in the clockwise direction except *nad1*, *nad4L*, *nad4,* and *nad5* (gene names underlined). The two ribosomal RNA genes are encoded by the L-strands (underlined). Transfer RNA genes are designated by single-letter amino acid codes, and those encoded by the H- and L-strands are shown outside and inside of the circular gene map, respectively. L1, L2, S1, and S2 denote *trnL* (*uur*), *trnL *(*cun*), *trnS *(*agn*), and *trnS *(*ucn)*, respectively. Two pairs of La-PCR primers [18] were used to amplify the complete mitogenome of *A. cinerea* into two overlapping fragments (from *cox1* to *cob* and from *cob* to *cox2*).

Two pairs of La-PCR primers [18] were used to amplify the complete mitogenome of *A. cinerea *into two overlapping fragments, *cox1*-*cob *(~9.5 bp) and *cob*-*cox2 *(~6 kb), as shown in Figure 1. La PCR amplifications were performed using Bio-Rad MyCycle Thermal Cycle (Bio-Rad, Hercules, USA) with 150 ng of genomic DNA, 2.5 *μ*L of 10 × LA PCR Buffer II (TaKaRa Bio Inc.), 5.0 mmol/L dNTP (2.5 mmol/L each dNTP), 62.5 mmol/L MgCl_2 _(25 mmol/L), 25 *μ*mol/L each primer (10 *μ*mol/L), 1.5 units of LA Taq polymerase (TaKaRa), and sterile distilled H_2_O to make up to 25 *μ*L reaction volume. The cycling protocol consisted of an initial denaturation step at 94°C for 2 min, followed by 40 cycles of denaturation at 94°C for 10 s, annealing at 45°C for 30 s, and elongation at 68°C for 8 min during the first 20 cycles and then an additional 20 s elongation per cycle during the last 20 cycles. The final elongation step was at 68°C for 7 min. LA-PCR products were purified with DNA Gel Purification Kit (U-Gene) after separation by electrophoresis in a 1.0% agarose gel.

Sub-PCR primers were designed based on the comparison of twelve hemimetabolous insect sequences recorded in GenBank. The amplifications were performed with 50 ng of La PCR products, 2.5 *μ*L of PCR Buffer (TaKaRa), 3.0 mmol/L dNTPs (2.5 mmol/L each dNTP), 62.5 mmol/L MgCl_2_, 15–50 *μ*mol/L each primer, 1.5 units of TaKaRa Taq polymerase (TaKaRa), and sterile distilled H_2_O up to 25 *μ*L reaction volume. The cycling protocol consisted of an initial denaturation step at 94°C for 2 min, followed by 25–30 cycles of denaturation at 94°C for 10 s, 40–50°C annealing for 30 s, and 72°C elongation for 1-2 min. The final elongation step was at 72°C for 7 min. The Sub-PCR products were purified by DNA Gel Purification Kit (U-Gene).

The Sub-PCR fragments were sequenced directly or cloned into TaKaRa pMD 18-T Vector (TaKaRa). All products were sequenced in both directions with the ABI PRISM 3100-Avant Genetic Analyzer with the sub-PCR primers and two vector-specific primers.

### 2.3. Data Analysis

We used the Staden package [19] for sequence assembly and annotation. Each gene was identified by sequence comparison with the mitochondrial sequence of *Locusta migratoria migratorioides *(X80245). For mitogenome comparative analysis, we downloaded 20 additional complete Orthoptera mitogenomes sequences from GenBank (Table 1). Homologous sequences for each gene were initially aligned using Clustal X [20], and further analyzed by MEGA version 4.0 [21]. 

The initial alignments of tRNA and rRNA genes were manually corrected for obviously misaligned positions in BioEdit 7.0.0 [34]. To infer secondary structures, we used a commonly accepted comparative approach [35, 36]. Briefly, we defined a compensatory change as two substitutions occurring sequentially that maintained base pairing in a given position of a helix. The observation that two or more Watson-Crick (or G • U) interactions at the same location in a putative helix indicated selection to maintain base pairing and thus supported the helical model [7]. Evidence from consistent and compensatory substitutions (CCSs) gave more concrete measurement of the length of tRNA arms. We used the secondary structure model of the *Drosophila melanogaster* mitochondrial *rrnL* and the *Chorthippus parallelus* and *Drosophila virilis* mitochondrial *rrnS* molecules [35] to search for conserved sequence motifs that can be associated with conserved structural elements. The initial screening for conserved structural sequence motifs facilitated the subsequent analysis of secondary structural elements in more variable parts of the molecule. By searching for CCSs, we established the most likely secondary structures for the more variable portions of the rRNA molecules. Additionally, the inferred secondary structures were validated by using the folding algorithm in the software RNAalifold [37]. The default settings were used to predict consensus structures in RNAalifold. Except for the standard Watson-Crick base pairs and noncanonical G • U interactions, noncanonical base pairings proposed in other models were all observed in our study. The conventional numbering system established in the CRW Site [35] was used if a potential homology could be established by sequence similarity and/or structural position. In comparison, consecutive numbering was used when structural homology was ambiguous. Secondary structures were drawn using the software RnaViz 2.0 [38]. Conserved stem-loop structure of A+T-rich region in some species of Orthoptera also establishes by CCSs. 

The complete mitochondrial genome sequence of the *A. cinerea* was deposited in GenBank with the accession number GU344100. 

## 3. Results and Discussion

### 3.1. Genome Organization and Composition

The length and the average AT content of the complete mitochondrial sequence of *A. cinerea* is 15, 599 bp and 76.07%, respectively, well within the range of Orthoptera (Table 1). It displays a typical gene composition found in insect mitogenomes: 13 PCGs, 22 tRNA genes, 2 rRNA genes and an A+T-rich region. Besides the A+T-rich region, 17 noncoding regions are present in *A. cinerea *mitogenome, comprised of a total of 80 nucleotides. Overlaps ranging from 7 to 8 bp span over 4 regions (Table 2). 

The orientation and gene order of the *A. cinerea* mitogenome (Figure 1) are identical to that of *L. migratoria* [24], exhibiting a translocation from the ancestral *trnK*/*trnD* to the derived *trnD*/*trnK*. Previously, this translocation was proposed and subsequently confirmed as a synapomorphy for Caelifera [14, 17, 18, 23–26, 28–33]. Furthermore, the duplicated *trnL *(*uur*) initially identified in *T*.* neglectus * [17] may serve as a potential molecular synapomorphy characteristic of a subgroup within Rhaphidophoridae. The translocation of *trnN*-*trnE*-*trnS* to *trnE*-*trnS*-*trnN* in *T. emma* has been reported [30], and appeared to be one of the most common changes in *Drosophila *as the result of sequence inversion of these tRNA clusters [39]. Future research will determine whether this rearrangement is a potential autapomorphy of this cricket or occurs at higher taxonomic level.

**Figure 2:**
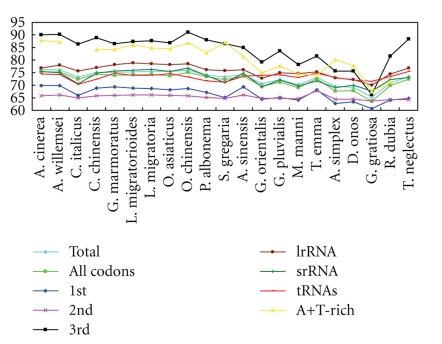
A+T content in different regions of the 21 Orthoptera mitogenome. Due to the partial A+T-rich regions of *C. italicus* and *T. neglectus*, we excluded them from the analysis.

The highest AT content was observed in the A+T-rich region and the third codon position which are both under the lower selection pressure. As the expectation, the first and second codon positions have the less A+T base position bias than other mitogenome regions. Although the A+T-rich region is hypervariable, it is not necessarily the most variable region in the genome in terms of nucleotide substitution [13, 40]. In this paper, the A+T content of the A+T-rich region is always lower than that of the third codon position of PCGs (Table 1 and Figure 2). The concentrations of adenine and thymine of *rrnL* molecular are higher than that of *rrnS*, PCGs and the whole genome slightly. The curves that are representatives of PCGs and whole genome are very close. In Orthoptera, the A+T contents of ensiferans are lower than those of caeliferan but have higher difference among the species, especially in the regions which have high A+T content. Nevertheless, tRNA and the second position of PCGs have the relative constant A+T concentration in orthopterans, indicating that they are structurally or functionally more constrained. 

### 3.2. Protein Coding Genes and Codon Usage

A typical ATN start codon was observed in eleven of the *A. cinerea *PCGs (Table 1). We assigned Ala (GCU) and Lys (AAA) to the *nad5* and *cox1 *gene as start codon, respectively. Conventional termination codons (TAA and TAG) were observed in most of the putative protein sequences except the genes of *cox2*, *nad2,* and *nad5* with incomplete termination codon T or TA- tRNA (Table 1). 

Excluding the termination codons, the 13 PCGs in the *A. cinerea* mitogenome comprise of 3721 codons in total. The codon usage and the relative synonymous codon usage (RSCU) values are summarized in Table 3. The most frequent amino acids in the PCGs of *A. cinerea* are leucine (13.52%), isoleucine (10.70%), serine (9.87%), and phenylalanine (9.50%).

### 3.3. Transfer RNA and Ribosomal RNA Genes

#### 3.3.1. tRNA Genes

The lengths of *A. cinerea* 22 tRNA genes range from 64 bp to 71 bp. The predicted secondary structures of tRNAs are shown in Figure 4. Most of the size variation among tRNAs stemmed from the length variation in the arm and loop of TΨC and the loop of DHU.

**Figure 3:**
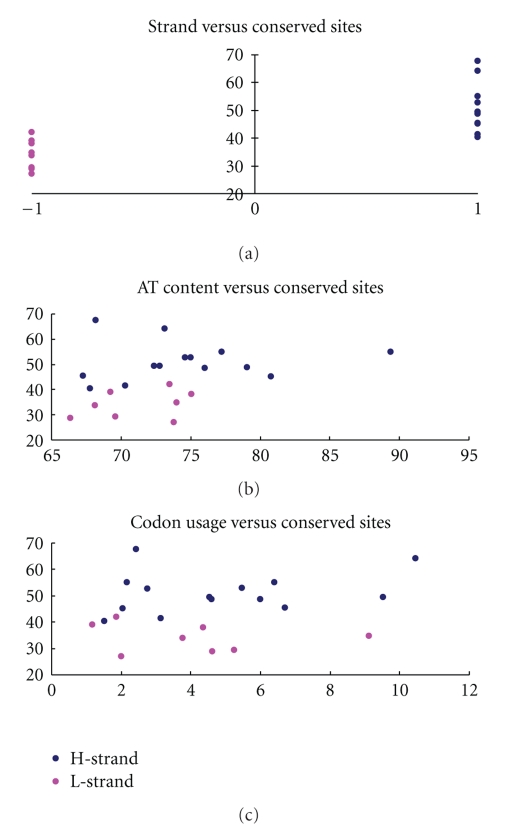
Coding strand versus conservation sites%, the average A+T content versus conservation sites% and codon usage% versus conservation sites% in the Orthoptera mitogenomes. The X-axis provides coding strand, the average A+T content of each tRNA and the average percent of codon usage values, while the Y-axis provides the percent of the conservation sites of each tRNA. Points referring to H- and L-strand tRNAs are shown using blue and pink colours, separately. On the X-axis of “coding strand versus conservation sites%”, 1 is assigned to tRNAs encoded by H-strand and −1 is assigned to the L-strand.

All tRNAs from 21 orthopterans have the typical clover leaf structure except for *trnS* (*agn*) [22, 25, 26, 28–33]. The percent of the conservation sites of each tRNA, coding strand, the average A+T content of each tRNA, and average percent of codon usage were calculated for 21 mitogenomes of Orthoptera and are presented in Figure 3. The tRNAs encoded by the H-strand generally contain more conservation sites than those encoded by the L-strand. The conservation of tRNA genes was not associated with the frequency of codon usage and A+T content.

All tRNAs genes contain a 7-bp amino acid acceptor (AA) stem, where most nucleotide substitutions are compensatory. However, noncanonical interactions likely contribute to the full stem structure especially at the fifth or sixth couplet of certain tRNAs. For example, U · U or C · C pairs were found at the sixth couplet of *trnQ* in most Caelifera species. Likewise, in Caelifera, noncanonical A · G and A · A pairs were observed in *trnW *and* trnD *at the fifth couplet. Furthermore, U · U pairs are located at the sixth couplet in *trnS *(*ucn*) of Ensifera, and U · U or C · C pairs at the sixth couplet in *trnA* of orthopteran. *Acrida* sequences share a cytosine insertion after the fifth couplet, potentially as a molecular synapomorphy for this genus. Primary sequences of this helix are highly conserved in *trnM* and *trnT*. 

The anticodon (AC) stem (5 bp) and the loop (7 bp) are both conserved in all tRNAs genes except for *trnG* of *T. emma*, which contains a distinct loop and two A · G pairs at the second and third couplets. Noncanonical interactions are also present in the AC stem, especially at the first couplet, including *trnM*, *trnW*,* trnK*, *trnR*, and *trnL *(*cun*). There is a conserved uracil before the anticodon in the AC loop. 

Except for *trnS *(*agn*), the length of DHU is 3- or 4-bp as established by CCSs, and relatively consistent for each tRNA. Primary sequences of the DHU stem of *trnI*, *trnM*, *trnW*, *trnD*, *trnE*, *trnT* are conserved in the referenced taxa. The loop of DHU varies among the tRNAs of orthopterans except in *trnQ* (5 bp) and *trnA *(4 bp). The second *trnL *(*uur*) copy of *T*.* neglectus* [17] differs from others in the primary sequence of the DHU stem and loop. In addition, *L*.* migratoria* and *O. chinensis* have an insertion after the second couplet of *trnH*.

The lengths of the TΨC arm range from 3-bp to 6-bp and the loop also varies among the tRNAs. Among the 22 tRNAs, 14 tRNAs contain a variable (V) loop of constant length, most commonly 4 bp. 

Except* trnS *(*agn*), the spacing nucleotides between the AA and DHU stems are predominantly nucleotides “UR”. Only one nucleotide separates the DHU and AC stems, except for *trnG* of *G. orientalis,* and *trnH* of Caelifera. *T. emma* has an insertion between the TΨC and AA stems of *trnG* as well as *trnL *(*cun*) of *P. albonema*, whereas there is no interval between these two stems in other tRNAs.

#### 3.3.2. rRNA Genes

We derived a secondary structure model of the *rrnS* and *rrnL* from 21 Orthoptera taxa using a comparative approach. The derived secondary structures closely resemble those from other insects on CRW, thus confirming the majority of previously proposed base pair interactions in the rRNA molecules.

The secondary structure of the *A. cinerea rrnS* is presented in Figure 5(a) as a representative of 21 orthopterans. It consists of 782 nucleotides and 28 helices. Similar to the secondary structure of small ribosomal RNA subunits in prokaryotes, the secondary structure of insect *rrnS* is subdivided into four principal domains (labeled I, II, III, and IV) with reduction of certain helices [8]. Domains I and II are less sequenced due to the use of variable and less universal primers. Domains III and IV are the most conserved regions of *rrnS*, routinely used in insect systematic studies as molecular markers.

Domain I contains 9 helices. The primary sequences of helix 17 and the distal part of helix 511 are conserved, whereas most of the remaining helices in domain I were established from CCSs. U · U pairs at the fifth couplet preserve a 5-bp helix 9 as proposed in other models [12, 35, 41]. Helices 27 and 39 form in all the taxa, although the hydrogen bonds are always disrupted in these two helices. Comparative analysis suggested eight couplets of helix 47 in Caelifera, and the initial two couplets are disrupted in most of the Ensifera taxa except *Gryllotalpa*. The single nucleotide bulges of helices 47 and 367 are conserved, often serving as sequence anchor in sequence alignment. The distal part of helix 511 is conserved among orthopteran; in contrast, the couplets of the proximal part are neither conserved nor covaried. Compared with the *E.coli* model, the region enclosed by helix 47 has a significant reduction in orthopteran, too variable for sequence alignment and general model construction. Previously, Mfold analysis [42] suggested two helices in this region of Caelifera, numbering helices 48 and 49 in Figure 5(a). However, it is difficult to draw a similar universal structure for the referenced sequences of Ensifera.

Domain II displays five helices. Helix 567 contains three base pairs established by CCSs. Similar to the *C. parallelus* model, most taxa of Caelifera have a 4-bp helix 577; in comparison, there are two additional couplets at the distal end of Ensifera. Helix 673 in almost all referenced sequences have two couplets and a 6-bp loop; however, the majority of the proximal part is less conserved unless in the same genus. RNAalifold analysis [43] indicated five nucleotide interactions (at position 215 : 219 to 260 : 264 in the 12S rRNA of* A. cinerea*) for Caelifera. The distal part of helix 769 is the most conserved region in domain II, encompassing the universal primer SR-N-14588. Six other base pairs likely reside at the base of helix 769. Nucleotides undergo covaried substitutions at the first three base pairs of helix 885. As in the *C. parallelus* model, we propose four couplets for the distal extension, although there are usually noncanonical interactions at the fourth and fifth couplets (350 : 362 and 353 : 359) of helix 885. 

The secondary structure of domain III has been demonstrated in many insect taxa [8, 11, 41, 44, 45]. The structure of this domain in this study is based on the *C. parallelus *model on CRW with min or difference such as the two additional couplets at the end of helix 921 as well as another conserved base pairing at the beginning of helix 944.

Helices 1399 and 1506 at the 3′ end of *rrnS* molecules are both conserved, and the constructed secondary structures are highly concordant with the *C. parallelus *model. Previously, the enlarged loop of helix 1399 was shown in Zygaenidae *Himantopterus dohertyi* and *Somabrachys aegrota *[12]. The loop of helix 1399 in Caelifera is substantially larger than those of moths (Figure 5(a)), potentially indicative of an autapomorphy of this insect group. The enlarged region after the thirteenth couplet usually starts with a conserved motif “AU” and ends by an adenine. About six couplets and a symmetrical bulge have been proposed to consist of the enlarged region in *C. parallelus*. However, since our data do not support this hypothesis, studies of additional sequences from Caelifera are needed to clarify this issue.

The *rrnL* of *A. cinerea *is 1316 bp in length and divided into six domains (labeled I, II, III, IV, V and VI), each separated by a single stranded region [41]. Domain III is absent in arthropods mitochondrion (Figure 5(b)). The majority of structural and phylogenetic studies had focused on the 3'-half of the *rrnL* molecule [7, 46–48], corresponding to highly conserved domains IV and V (Figure 5(b)). Due to relative high variability and few applicable primer sets [1], domains I, II, and VI are seldom used in secondary structure prediction and molecular phylogenetic studies [41]. 

Compared to the* E.coli* model, considerable degeneration in domain I of Orthoptera leads to only five remaining helices. This initial region of the *rrnL *molecule is highly variable and difficult to align. Consistent with the *D*. *melanogaster* model [35], two stems (helices 183 and 235) are hypothesized before helix 461. Comparative sequence analysis has established the second, third, and fourth couplets of helix 235, but convincing evidence for a 2-bp helix 183 in Orthoptera is still missing. Although a few noncanonical interactions U · U are found at the second couplet of helix 461 in Caelifera, it is supported by CCSs in the taxa of Ensifera. Nucleotides surrounding helices 461 and 533 are highly conserved, with helix 563 as the most conserved helix of domain I both in primary sequence and secondary structure. 

Domain II is not well conserved; nevertheless, most of the helices are established by compensatory changes including the long-distance pairing helices 579 and 812. Hydrogen bonds of the last two base pairs of helix 671 and the initial two couplets of helix 946 are disrupted in Caelifera, but remain intact in Ensifera. Regions between helices 822 and 946 and helices 946 and 812 are extremely variable, exhibiting distinct shapes in different models [35, 41, 49]. A 4-bp helix 991 is predicted according to CCSs. The distal part of helix 1057 is constant in Orthoptera species. The internal bulge of helix 1087 is unstable in certain Ensifera species. The primary sequence and secondary structure of helix 1196 are extremely variable in Orthoptera except for the initial couplet as confirmed by CCSs.

Domain VI contains 3 helices. The distal part of helix 2646 is extremely conserved. Despite certain noncanonical interactions or mismatches, the 7 base pairs of helix 2646 are validated by CCSs. In most of the taxa, a 5-bp helix 2675 terminated with a variable loop is predicted, whereas the structure of helix 2735 is unclear.

### 3.4. A+T-Rich Region

The largest noncoding region of insect mtDNA, called the “AT-rich region” due to its high AT content, is considered to be involved in the regulation of mtDNA transcription and replication [1]. It is often unclear whether these “control elements” are homologous between distantly related animal or have arisen from various noncoding sequences independently in separate evolutionary lineages due to the low sequence similarity except among closely related animals [2].

As with other Orthoptera species, the A+T-rich region of *A. cinerea *is located between *rrnS* and *trnI* (Figure 1 and Table 1). It is 784 bp in length and 87.88% A+T content, both within the range of Orthoptera, and apparently contains no repeat region. Among the 21 orthopterans studied here, the length of the A+T-rich region ranges from 70 bp in *R. dubia* to 1401 bp in *O. asiaticus *(Table 1). The length differences among closely related taxa are mainly caused by the variation in the size and copy number of repeat units [50].

The Orthoptera sequences studied here belong to four different superfamilies, including 12 Acridoidea, 1 Pyrgomorphoidea, 5 Grylloidea, and 5 Tettigoniidea. The first two groups belong to Caelifera, and the remaining groups belong to Ensifera. The control region between the two *Acrida* species is highly similar, and the percentage of identity nucleotide is 97.07%. The main difference between the two subspecies of *L. migratoria* is the copy number of repeat units.

 In Orthoptera, large repeat regions have been reported in *X93574 Chorthippus parallelus * [50] and* X15152 Gryllus firmus *[51] as well as in the mitochondrial genomes of *L. migratoria *[24], *G. marmoratus* [23], *O. asiaticus* [23]*, L*.* m. migratoria*, *T*.* emma* [30], and *G. gratiosa* [32]. Most of the tandemly repeated sequences were found at the end next to the *rrnS* and the first repeat begins with a 12 (in *C. parallelus*) ~64 (in *G. gratiosa*) nucleotide extension at the *rrnS* (Table 4). However, in *O. asiaticus*, two different repeat units are present on either end of the A+T-rich region. The final repeat at the 3′ end usually has more sequence variations than the others. In addition to strong conservation in the same sequence, the repeat units also show little variation in subfamily Oedipodinae (Table 4). Although the repeat units of *G*.* firmus* and *T*.* emma *show low sequence identities (Table 4), the shared dyad symmetric sequence 5′-GGGGGCATGCCCCC-3′ may be a conserved motif in this subfamily.

A potential stem-loop structure, potentially involved in replication initiation, is located at the central region near the *trnI *gene of *L. migratoria*, and easily distinguished from the repeated sequence [52]. Besides desert locust *S. gregaria* and the meadow grasshopper *C. parallelus * [50], a stem-loop structure, 16 bp or longer, also exists in the same position in all of the taxa from Caelifera. Nucleotides of this region are almost identical except for the distal three base pairs as revealed by compensatory substitutions (Figure 6). The flanking regions, including “TATA” on the 5′ end and “G (A)_n_T” on the 3′ end, are also conserved in Caelifera except *O. chinensis* and *A. sinensis*. Other conserved structural elements [13, 50] were also found in the referenced species of Caelifera, except for the long polythymine stretch often interrupted by other nucleotides such as cytosine. *Acrida* sequences lack the >4 bp T-stretch. Rather, the motif “TATTTwATryAyAAA” adjacent to the tRNA^Ile^ is more conserved in the Caelifera taxa (Figure 6).

**Figure 4:**
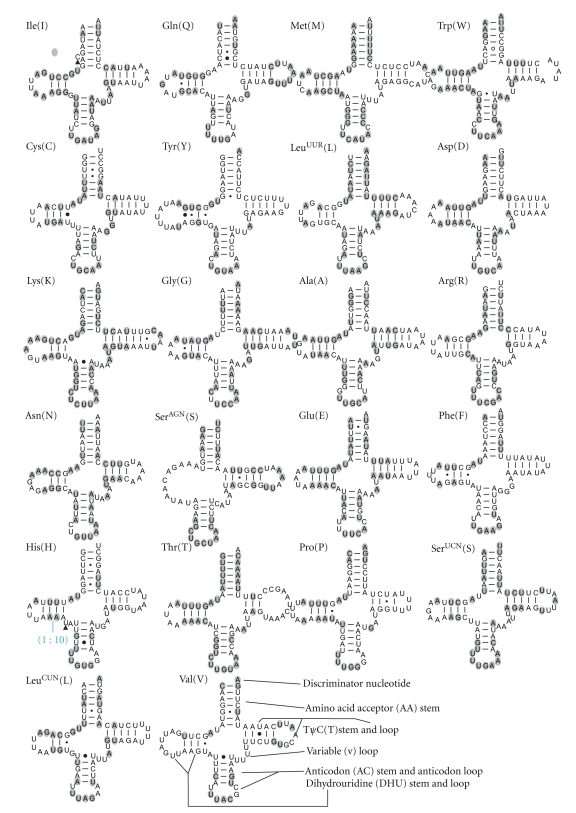
Inferred secondary structure of 22 tRNAs in the *A. cinerea* mitogenome. The tRNAs are labeled with the abbreviations of their corresponding amino acids. Positions conserved across all sampled Orthoptera taxa are circled in grey. Base pairing is indicated as follows: standard canonical pairs by lines (C–G, G–C, A–U, and U–A); wobble G *·* U pairs by dots (G · U); A · G pairs by open circles (A ∘ G); other noncanonical pairs by filled circles (e.g., C ● A). Blue tags and solid triangle (▲) indicate insertions relative to the reference sequence. All secondary structures were drawn using the program RnaViz 2.0 [38] with manual adjustment.

**Figure 5:**
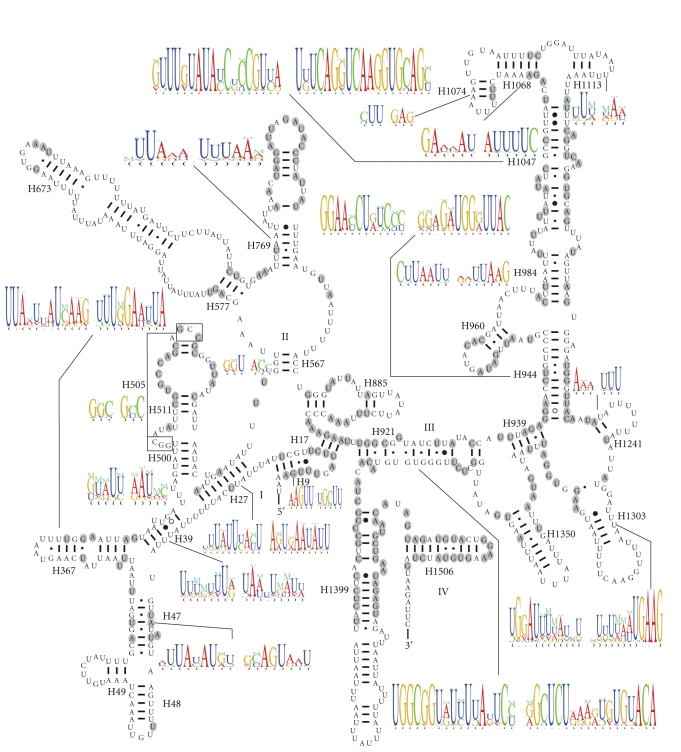

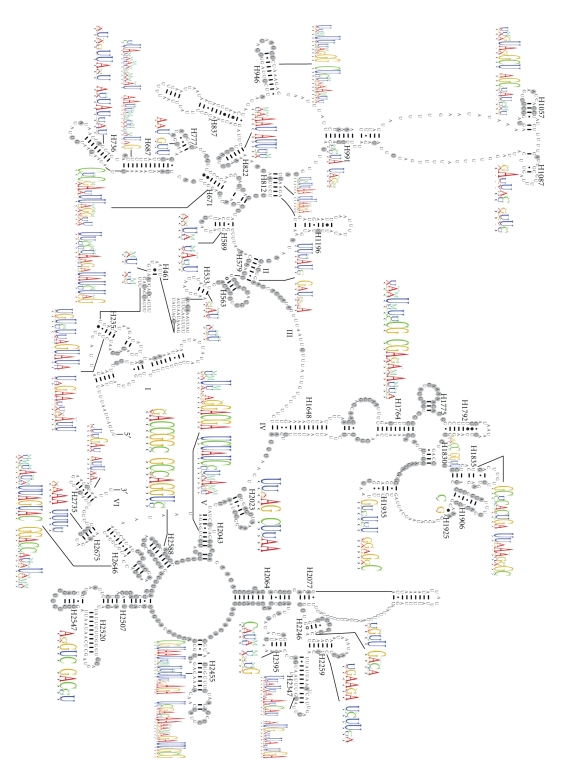
The secondary structure model of the mitochondrial rRNAs from *A. cinerea*. The helix numbering system has been described [35], except for the variable region enclosed by Helix 47 in rrnS. Positions conserved among all sampled taxa of Orthoptera are circled in grey. The consensus sequence, relative frequency of nucleotides, and information content of selected helices are displayed by structure logos (height of a nucleotide symbol is proportional to its frequency; letter M indicates the amount of mutual information). Roman numerals specify domains I–IV. (a) rrnS (b) 5′ half of rrnL. (c) 3′ half of rrnL. See Figure 4 legend for explanation on base pair symbols and software used to construct structure diagrams.

**Figure 6:**
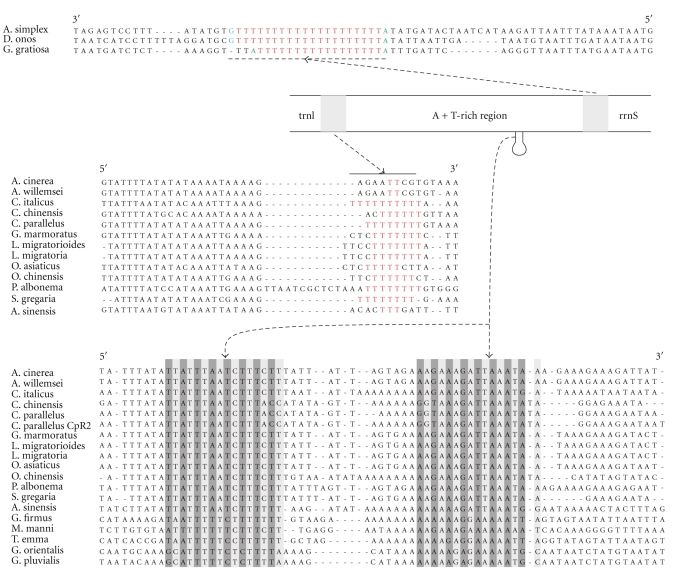
The nucleotide sequences around the T-stretches and stem-loop of Orthoptera mtDNA.

Previously, it was proposed that a sequence segment in each repeat unit forms a stem loop structure with homologous to those found in *Drosophila* and *S. gregaria*/*C. parallelus*. If the stem-loop structure for replication initiation is included in the repeated sequence, the same structure may also exist in the closely related *T. emma* mtDNA sequence. However, in *T. emma*, the proposed stem-loop [50] in each repeat unit contains more mismatches between base pairs. In addition, *M. manni*, another Gryllidae species, lacks a large tandem repeat in A+T-rich region, suggesting that additional sequences may be involved in replication initiation. Two adjacent nucleotide stretches were found in the sequences of *G. firmus, T. emma and M. manni*, with a T-stretch interrupted by C located upstream of an A-stretch interrupted by *G. firmus.* These two stretches may form a 16-bp stem and loop structure similar to that of Caelifera, coincidently located at the corresponding position except for *G. firmus* (Figure 4). In *Gryllotalpa*, a similar stem-loop structure was also detected. Furthermore, the structure was well established by CCSs in the Grylloidea superfamily. 

In conclusion, the stem-loop predicted in this study is likely to be involved in replication initiation in the taxa of Caelifera and Grylloidea. In contrast with these two taxa, detection of the conserved stem-loop structure in the Tettigoniidae is more difficult. Three available complete genomes in Tettigoniidae (*A. simplex* [14], *D. onos* and *G. gratiosa*) exist a common feature with a long T-stretch (>17 bp) *next to rrnS* on the H-strand, bounded by a purine at either end.

## 4. Conclusions

The mitogenome of *A. cinerea* displays characteristic gene content, genome organization, nucleotide composition, and codon usage found in other Caelifera mitogenomes. Comparison of all available 21 orthopteran mitogenomes provides us more information about the evolution of mitogenomes in this insect group.

Comparison of tRNAs sequences from Orthoptera revealed that the conservation of tRNA genes was not associated with the frequency of codon usage but rather with the coding strand. The tRNAs encoded by the H-strand appear more conserved than those by the L-strand. All tRNAs form the typical clover-leaf structure except *trnS* (*agn*). Most of the size variation among tRNAs stemmed from the length variation in the arm and loop of TΨC and the loop of DHU. 

The secondary structure models of the* rrnS* and *rrnL* from 21 Orthoptera taxa were predicted using the comparative approach. The derived secondary structures closely resemble those from other insects on CRW except a considerably enlarged loop of helix 1399 of *rrnS* in Caelifera, thus confirming the majority of previously proposed base pair interactions in the rRNA molecules. 

In the A+T-rich region of Orthoptera, tandem repeats are not only conserved in individual mitogenome but also show conserved sequence blocks in the same subfamily. Conserved stem-loop structures, potentially involved in replication initiation, were found at the similar position within the A+T-rich region of all Caelifera and Grylloidea mitogenomes. A long T-stretch (>17 bp) with conserved stem-loop structure next to *rrnS* on the H-strand, bounded by a purine at either end, exists in the three species from Tettigoniidae.

## Figures and Tables

**Table 1:** Characteristics of the 21 complete mitochondrial genome sequences of Orthoptera including *A. cinerea*. ^a^Termination codons are excluded. *Incomplete A+T-rich region.

Taxon	Accession number	Total		PCG^a^					lrRNA		srRNA		tRNAs		A+T-rich region		References
		bp	%A+T	Number of codons^b^	%A+T				bp	%A+T	bp	%A+T	bp	A+T%	bp	A+T%	
					Total	First	Second	Third									
**Caelifera**																	
Acridoidea																	
* Acrida cinerea*	GU344100	15599	76.07	3720	75.28	69.84	65.86	90.13	1316	76.82	782	75.45	1474	74.41	784	87.88	This study
* Acrida willemsei *	EU938372	15601	76.22	3716	75.37	69.81	66.04	90.26	1314	78.08	718	74.93	1476	74.32	848	87.26	[17]
* Calliptamus italicus *	EU938373	15675	73.22	3717	72.39	65.94	64.92	86.31	1322	75.64	801	70.54	1485	70.44	^*∗*^	^*∗*^	[17]
* Chorthippus chinensis *Tarb	EU029161	15599	75.11	3713	74.50	68.81	65.72	88.96	1313	77.00	843	74.73	1477	72.44	721	84.05	[22]
* Gastrimargus marmoratus *	EU513373	15924	75.18	3725	73.91	69.29	65.91	86.52	1322	78.14	831	75.69	1470	74.83	1061	84.26	[23]
* Locusta migratoria migratorioides*	X80245	15722	75.33	3713	74.08	68.84	66.04	87.37	1314	78.84	827	75.94	1469	74.00	875	85.94	[24]
* Locusta migratoria migratoria *	EU287446	16053	75.53	3713	74.21	68.73	66.17	87.72	1316	78.50	834	76.26	1471	73.96	1189	84.86	(Xiao, 2007, unpublished)
* Oedaleus decorus asiaticus *	EU513374	16259	75.39	3716	73.80	68.20	65.90	86.80	1318	78.15	831	75.45	1471	74.64	1401	84.51	[23]
* Oxya chinensis*	EF437157	15443	75.89	3727	75.16	68.63	65.71	91.15	1317	78.59	848	76.77	1475	73.29	562	86.83	[25]
* Phlaeoba albonema *Zheng	EU370925	15657	74.11	3721	73.47	67.16	65.17	88.09	1312	76.14	844	73.82	1486	71.74	728	82.97	[26]
* Schistocerca gregaria gregaria *	GQ491031	15625	73.18	3718	72.15	65.14	64.77	86.55	1320	75.83	813	71.09	1477	71.23	762	87.01	[27]
Pyrgomorphoidea																	
* Atractomorpha sinensis *	EU263919	15558	74.29	3711	73.52	69.39	66.15	85.02	1311	76.20	819	74.85	1462	73.60	778	81.36	[28]
**Ensifera**																	
Grylloidea																	
* Gryllotalpa orientalis *	AY660929	15521	70.49	3705	69.44	64.35	64.83	79.14	1247	72.81	719	69.40	1447	73.88	920	74.89	[29]
* Gryllotalpa pluvialis *	EU938371	15525	72.20	3689	71.14	65.06	64.81	83.55	1236	74.92	783	72.03	1447	74.15	867	77.74	[17]
* Myrmecophilus manni *	EU938370	15323	70.18	3677	68.97	64.10	64.62	78.19	1252	74.60	734	69.75	1433	73.13	789	74.52	[17]
* Teleogryllus emma *	EU557269	15660	73.12	3700	72.56	68.22	67.84	81.62	1293	75.25	812	71.67	1456	74.86	940	73.94	[30]
Tettigoniidea																	
* Anabrus simplex *	EF373911	15766	69.44	3725	67.62	62.74	64.40	75.70	1312	72.87	785	68.92	1458	73.05	987	80.14	[14]
* Deracantha onos *	EU137664	15650	69.24	3729	67.78	63.37	64.39	75.57	1301	72.25	858	70.05	1467	71.98	815	77.79	[31]
* Gampsocleis gratiosa *	EU527333	15929	65.31	3729	63.56	60.63	63.80	66.24	1317	70.01	848	67.69	1447	71.53	1111	67.42	[32]
* Ruspolia dubia *	EF583824	14971	70.86	3728	69.90	64.00	64.19	81.52	1302	74.42	882	72.22	1470	73.47	70	71.43	[33]
* Troglophilus neglectus *	EU938374	15810	73.37	3727	72.47	64.82	64.26	88.33	1342	76.83	785	73.12	1531	75.38	*	*	[17]

**Table 2:** Organization of the *A*. *cinerea *mitogenome. ^a^Without stop codons. ^b^Numbers correspond to nucleotides separating each gene from the previous one; negative numbers refer to overlaps between genes. ^c^Complete stop codons are presumably added by polyadenylation which are represented by *trnX* after the T or TA.

Gene or region	Start	End	Strand (Plus/Minus)	Length^a^	Intergenic nucleotides^b^	Start	Stop^c^
*trnI *	1	67	Plus	67	0		
*trnQ*	68	136	Minus	69	3		
*trnM *	140	208	Plus	69	0		
*nad2*	209	1229	Plus	1020	0	ATG	T-*trnW *
*trnW *	1230	1296	Plus	67	− **8**		
*trnC *	1289	1352	Minus	64	6		
*trnY *	1359	1427	Minus	69	− **8**		
*cox1*	1420	2959	Plus	1539	0	**AAA**	T-*trnL *
*trnL *(*uur*)	2960	3024	Plus	65	2		
*cox2*	3027	3708	Plus	681	0	ATG	T-*trnD *
*trnD *	3709	3773	Plus	65	2		
*trnK *	3776	3846	Plus	71	14		
*atp8*	3861	4022	Plus	159	− **7**	ATA	TAA
*atp6*	4016	4693	Plus	675	3	ATG	TAA
*cox3*	4697	5488	Plus	789	2	ATG	TAA
*trnG *	5491	5557	Plus	67	0		
*nad3*	5558	5911	Plus	351	1	ATT	TAA
*trnA*	5913	5979	Plus	67	3		
*trnR*	5983	6046	Plus	64	2		
*trnN*	6049	6114	Plus	66	0		
*trnS *(*agn*)	6115	6181	Plus	67	0		
*trnE *	6182	6247	Plus	66	1		
*trnF *	6249	6313	Minus	65	0		
*nad5*	6314	8040	Minus	1725	6	**GCU**	TA-*trnF *
*trnH *	8047	8112	Minus	66	3		
*nad4*	8116	9450	Minus	1332	− **7**	ATG	TAG
*nad4L*	9444	9737	Minus	291	0	ATG	TAA
*trnT *	9738	9806	Plus	69	0		
*trnP *	9807	9871	Minus	65	0		
*nad6*	9872	10393	Plus	519	6	ATG	TAA
*cob*	10400	11539	Plus	1137	2	ATG	TAA
*trnS *(*ucn*)	11542	11611	Plus	70	21		
*nad1*	11633	12577	Minus	942	3	ATG	TAG
*trnL *(*cun*)	12581	12645	Minus	65	0		
*rrnL *	12646	13961	Minus	1316	0		
*trnV *	13962	14033	Minus	72	0		
*rrnS*	14034	14815	Minus	782	0		
A+T-rich region	14816	15599	Minus	784	0		

**Table 3:** Codon usage of PCGs in the *A. cinerea* mitogenome. A total of 3720 codons were analyzed, excluding termination codon. n: frequency of each codon; RSCU: Relative Synonymous Codon Usage. *Stop codons.

Codon(aa)	n(RSCU)	Codon	n(RSCU)	Codon	n(RSCU)	Codon	n(RSCU)
UUU(F)	**296.0(1.68)**	UCU(S)	109.0(2.38)	UAU(Y)	147.0(1.71)	UGU(C)	37.0(1.72)
UUC(F)	57.0(0.32)	UCC(S)	9.0(0.20)	UAC(Y)	25.0(0.29)	UGC(C)	6.0(0.28)
UUA(L)	**356.0(4.25)**	UCA(S)	129.0(2.81)	UAA(^*∗*^)	0.0(0.00)	UGA(W)	87.0(1.78)
UUG(L)	36.0(0.43)	UCG(S)	3.0(0.07)	UAG(^*∗*^)	0.0(0.00)	UGG(W)	11.0(0.22)
CUU(L)	44.0(0.52)	CCU(P)	53.0(1.57)	CAU(H)	52.0(1.58)	CGU(R)	22.0(1.52)
CUC(L)	3.0(0.04)	CCC(P)	4.0(0.12)	CAC(H)	14.0(0.42)	CGC(R)	1.0(0.07)
CUA(L)	62.0(0.74)	CCA(P)	73.0(2.16)	CAA(Q)	53.0(1.66)	CGA(R)	35.0(2.41)
CUG(L)	2.0(0.02)	CCG(P)	5.0(0.15)	CAG(Q)	11.0(0.34)	CGG(R)	0.0(0.00)
AUU(I)	**364.0(1.83)**	ACU(T)	53.0(1.04)	AAU(N)	160.0(1.81)	AGU(S)	31.0(0.68)
AUC(I)	34.0(0.17)	ACC(T)	15.0(0.30)	AAC(N)	17.0(0.19)	AGC(S)	1.0(0.02)
AUA(M)	**256.0(1.75)**	ACA(T)	131.0(2.58)	AAA(K)	79.0(1.55)	AGA(S)	79.0(1.72)
AUG(M)	37.0(0.25)	ACG(T)	4.0(0.08)	AAG(K)	23.0(0.45)	AGG(S)	6.0(0.13)
GUU(V)	90.0(2.16)	GCU(A)	60.0(1.59)	GAU(D)	64.0(1.73)	GGU(G)	91.0(1.69)
GUC(V)	2.0(0.05)	GCC(A)	5.0(0.13)	GAC(D)	10.0(0.27)	GGC(G)	5.0(0.09)
GUA(V)	71.0(1.70)	GCA(A)	84.0(2.23)	GAA(E)	71.0(1.75)	GGA(G)	114.0(2.12)
GUG(V)	4.0(0.10)	GCG(A)	2.0(0.05)	GAG(E)	10.0(0.25)	GGG(G)	5.0(0.09)

**Table 4:** Tandem repetition of A+T-rich region in Orthoptera. ^a^Percentage of identity at the nucleotide level between the first repeat unit and others in the same mitogenome. ^b^Percentage of identity at the nucleotide level between GmR1 and LmR1. ^c^Percentage of identity at the nucleotide level between GmR1 and LmmR1. ^d^Percentage of identity at the nucleotide level between GmR1 and OaRa1. ^e^Percentage of identity at the nucleotide level between GfR1 and TeR1.

					Identity%^a^	
Taxonx	Repeat unit	position	Length (bp)	A+T%		
					Intra-spceies^a^	Inter-species
*C. parallelus*	CpR1	1–777	777	85.20		
	CpR2	778–1512	735	85.31	90.37	
*G. marmoratus *	GmR1	14813–14978	166	80.12		
	GmR2	14979–15144	166	80.12	100.00	
	GmR3	15145–15299	155	79.36	82.63.	
*L. migratoria*	LmR1	14797–14951	155	79.36		71.08^b^
	LmR2	14952–15097	146	75.34	87.10	
*L. migratoria migratoria*	LmmR1	14814–14969	156	79.48		73.49^c^
	LmmR2	14970–15124	155	80.00	99.36	
	LmmR3	15125–15279	155	80.00	99.36	
	LmmR4	15280–15401	122	82.79	78.21	
*O. asiaticus *	OaRa1	14810–14964	155	75.49		61.08^d^
	OaRa2	14965–15119	155	76.13	96.77	
	OaRa3	15120–15260	141	75.89	86.45	
	OaRb1	15460–15786	327	90.83		
	OaRb2	15787–16143	357	89.92	91.32	
*G. firmus *	GfR1	114–333	220	65.00		
	GfR2	334–553	220	64.55	99.09	
	GfR3	554–747	194	65.47	87.73	
*T. emma *	TeR1	14664–14850	187	67.38		54.22^e^
	TeR2	14868–15055	188	67.55	97.35	
*G. gratiosa*	GgR1	14755–14956	202	55.94		
	GgR2	14976–15177	202	57.92	98.02	

## Abbreviations

*atp6 *and atp*8*: Genes encoding for ATP synthase subunits 6 and 8
*cob*: Gene encoding for cytochrome oxidase *b *
*cox1-3*: Genes encoding for cytochrome *c* oxidase subunits I-III
*nad1-6* and *nad4L*: Genes encoding for NADH dehydrogenase subunits 1–6 and 4L
*rrnL* and *rrnS*: Genes encoding for the large and small subunits of ribosomal RNA
lrRNA and srRNA: Large and small subunits of ribosomal RNA
*trnX*: Genes encoding for transfer RNA molecules with the corresponding amino acid denoted by the one-letter code and anticodon indicated in parentheses (*nnn*) when necessary
tRNA-X: Transfer RNA molecules with the corresponding amino acids denoted by a one-letter code and anticodon indicated in parentheses (NNN) when necessary
PCG: Protein coding gene
CR: Control region
NCR: Noncoding region
bp: Base pair (s)
kb: Kilobases
nt: Nucleotide (s)
aa: Amino acid (s)
mtDNA: Mitochondrial DNA
PCR: Polymerase Chain Reaction.
